# Causative role of gut microbiota in non-alcoholic fatty liver disease pathogenesis

**DOI:** 10.3389/fcimb.2012.00132

**Published:** 2012-10-26

**Authors:** Anna Alisi, Sara Ceccarelli, Nadia Panera, Valerio Nobili

**Affiliations:** Liver Research Unit and Hepato-Metabolic Disease Unit, “Bambino Gesù” Children's HospitalIRCCS, Rome, Italy

Non-alcoholic fatty liver disease (NAFLD) is one of the most common causes of chronic liver disease worldwide (Milić and Stimac, 2012). NAFLD affects prevalently children and adults with particular risk factors including genetic susceptibility and inappropriate lifestyle (i.e., over-/mal-nutrition and physical inactivity). In fact, obesity, as well as some traits of metabolic syndrome, such as insulin resistance and dyslipidemia, are co-morbidities often associated to the presence of NAFLD (Vanni et al., 2010). In line with the increased obesity epidemics, epidemiological studies indicate that the estimated global prevalence of NAFLD ranges from 3–10% depending on age, sex, ethnicity, and risk factors. Interestingly, in obese children and adults this prevalence may raise up to 20–80% (Alisi et al., 2009; Vernon et al., 2011).

The term of NAFLD defines a series of hepatic pathologies that include the relatively benign steatosis that may, under the pressure of multiple triggering factors, progress to the more severe condition of non-alcoholic steatohepatitis (NASH), characterized by steatosis, necro-inflammation, and eventually fibrosis (Brunt, 2010).

The NAFLD development is still unclear, however, it is now largely accepted that, beside to the genetic background, the increased consumption of obesogenic foods may have a role in the NAFLD pathogenesis. In particular, diets enriched in fat and fructose may be steatogenic in two ways: favoring the occurrence of systemic insulin resistance closed to a dangerous accumulation of free fatty acid (FFA) in the liver; causing deposition of visceral fat and consequent hepatic insulin resistance responsible for steatosis development (Tilg and Moschen, 2008). Steatotic liver is susceptible to the action of next insults that may exacerbate steatosis and promote NASH. These NASH promoters include: imbalance of production/release of hormones derived from adipose tissue (adipocytokines) with consequent necro-inflammation, oxidative stress, activation of specific nuclear receptors activation, and fibrogenesis (Malaguarnera et al., 2009). In response to the systemic insulin resistance, pancreatic β-cells increase insulin hypersecretion accelerating liver fat accumulation and leading to NAFLD. Recently, it has been reported that also gut-liver axis may play a crucial role in this complex network of multiple interactions (Musso et al., 2010a). In fact, it has been suggested that the diet-dependent increase of gut microbiota products may influence intestinal permeability and activate molecular mechanisms of innate immune response, acting as possible inductor of necro-inflammatory lesions and severe fibrosis in NAFLD (Compare et al., 2012).

**Figure 1:**
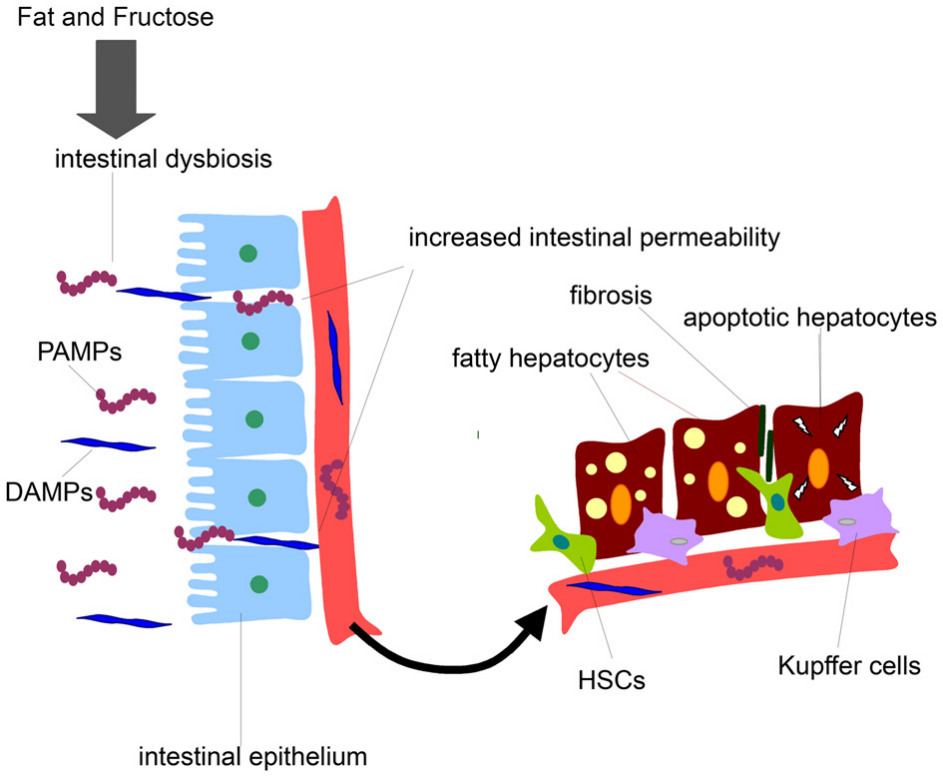
**Gut-derived product effects on liver cells during NAFLD development.** Diets enriched in fat and fructose may disrupt intestinal barrier, increase intestinal permeability to gut-derived products, and release of PAMPs and DAMPs into the systemic circulation. PAMPs and DAMPs may reach liver cells promoting firstly fat accumulation in hepatocytes and secondly fibrosis and liver cell damage.

The gut microbiota having an extensive cross-talk with the liver represents an important source of hepatotoxic factors comprising bacteria and bacterial products. In such as scenario the intestinal microbiota composition can be affected and dynamically altered by diet regimen, lifestyle, genetic background, antibiotic usage, etc (Compare et al., 2012). The gut microflora exerts various central functions, among which fermentation of dietary components eluding the digestion and protection against possible invading pathogens (Othman et al., 2008; Cani and Delzenne, 2009). Therefore, the maintenance of the integrity of the intestinal barrier is of crucial importance to preserve an healthy gut-liver axis. In fact, a derangement of the homoeostasis between bacteria and host and a qualitative and quantitative alteration of gut microflora lead to an increased intestinal permeability. This promotes bacterial and endotoxin translocation triggering a production of pro-inflammatory molecules and cytokines and metabolic disorders. Notably, the intestinal flora, comprised small intestinal overgrowth (SIBO), has been found altered in many chronic liver diseases. On this regard it has been shown that NAFLD patients have an increased intestinal permeability and SIBO (Miele et al., 2009). Several lines of evidence have shown that NAFLD can be affected in different way by gut microbiota. From a side, the microbiota can directly affect the quantity of calories recovered from intestinal contents influencing the body weight possibly preceding the obesity occurrence. Further, gut microbiota and related endotoxemia can be implicated in the development of insulin resistance involved in NAFLD pathogenesis by various mechanisms (Cani et al., 2007a,b; Musso et al., 2010b). Also, the intestinal integrity can be lost by the alteration of the tight junctions causing an increase intestinal permeability which leads to bacterial translocation and their products into the systemic circulation, which in turn reach the liver being correlated to NAFLD (Miele et al., 2009; De Gottardi and McCoy, 2011). The gut liver-axis is the way by which the bacteria and their potential hepatotoxic products (LPS, DNA, RNA, etc.) can easily reach the liver. These microbial compounds can be generally classified as pathogen-associated molecular patterns (PAMPs) while the endogenous products are distinguished in damage-associated molecular patterns (DAMPs). Interestingly, the serum levels of lipopolysaccharide (LPS), one of most studied PAMPs, are up-regulated in NASH patients with necro-inflammation and sever liver damage (Alisi et al., 2010). The final effect is the activation of the signaling cascade triggered by specific immune receptor resulting in the expression of pro-inflammatory cytokine genes including tumor necrosis factor (TNF)α and several interleukins (ILs) that may exacerbate hepatocyte damage (Figure 1).

The immune system has generated, along the evolution, a series of specific pattern recognition receptors (PRRs) comprising the Toll-like receptors (TLRs), which are evolutionary conserved type I transmembrane glycoproteins. The TLRs are the immune sensors of PAMPs and/or DAMPs initiating a signaling cascade leading to the activation of pro-inflammatory genes. Notably, each TLR has selectivity for specific PAMPs or DAMPs and can also differ from their proper localization (Alisi et al., 2011). In the liver, TLRs are expressed in many different cell types including Kupffer cells, hepatocytes, and HSCs (Schwabe et al., 2006) being the TLR4 the specific receptor for the bacterial endotoxin LPS, which is the key inducer of pro-inflammatory cytokines (as TNFα, IL-6, IL-8, IL-12, etc.) through the activation of the transcription factors NF-kB (nuclear factor kappa B), AP-1 (activating protein 1) and also LITAF (LPS-induced TNFα factor) in the liver (Alisi et al., 2011).

The gut colonization initiates at birth establishing a dynamic repertoire of gut microbiota during life that can be altered in several way giving rise to different inflammatory conditions possibly leading to more severe disturbances such as NAFLD. Many effort have been made to characterize which bacterial composition is more healthy to preserve the host from metabolic disorders and many progresses have been obtained in animal model on the study of the role of gut microbiota and accumulating evidence from human studies are available, although the examination along the time of the gastrointestinal bacteria composition is more difficult to assess. It has been extensively demonstrated that the proportion of intestinal microbiota is dependent on diet regimen. Interestingly, both in obese human and in mice the amount of the phyla Bacteroidetes and Firmicutes, which represent more than 90% of the totality of the gut microbiota, are altered (the first decreased and the second increased) (Eckburg et al., 2005). It has also been shown that a different balance of *Bifidobacterium* spp. and faecal S*taphylococcus aureus* results from the comparison between normal weight children and children which, at long last, can develop into overweight or obese subjects, suggesting a possible preceding condition that can foretell the future obesity occurrence (Kalliomäki et al., 2008). Further, the fat consumption can produce a wide amount of lipoprotein-containing chylomicrons which can guide the translocation of LPS toward other organs comprised the liver (Vreugdenhil et al., 2003). Interestingly, in a study in human choline-depleted diet it was described a correlation among the balance of *Gammaproteobacteria*/*Erysipelotrichi* classes and the occurrence of fatty liver (Spencer et al., 2011). Besides, in a murine models fed high-fat diet, the *Bifidobacterium* spp. administration determined an amelioration of the metabolic panel and a decrease of pro-inflammatory cytokines. Interestingly, the cytokine levels were augmented in parallel with LPS amount and inversely to *Bifidobacterium* spp. totality (Cani et al., 2007a,b). Confirming the important correlation among gut microbiota alteration and diet, various studies have reported that an high-fat diet increases the circulating LPS level amplifying the expansion of bacteria releasing LPS (Cani et al., 2007a,b). Moreover, an high-fat diet can cause a suppression of *Eubacterium rectale*, *Clostridium coccoides*, and *Bifidobacterium* spp (Cani and Delzenne, 2009). On the light of this evidence many probiotics have been tested in order to restore a balance in the altered gut microflora. *Lactobacillus* and *Bifidobacterium* spp are the most used having diversified beneficial results in the metabolic derangements such amelioration of dysplidemia, reduction of both total, LDL and VLDL cholesterol, decrease of triglycerides which overall are diversified effects depending on the bacterial species (Xiao et al., 2003; Tannock, 2004; Larkin et al., 2009). Noteworthy, the alteration of “healthy microbiota” is only one side of the complex interactions of the gut-liver axis. The disbiosys is a fraction of the causes leading to metabolic and gastrointestinal disturbances. All along, it is emerging a number of studies correlating the reciprocal interactions occurring among bacteria, viruses, eukaryotes, and in turn their communication with the host immune system. Indeed, the sequencing project Human Microbiome Project and the Earth Microbiome Project would be valid instruments to reach a wider and comprehensive overall view in order to better understand the interdependence between the host microbiota and the numerous disorders and diseases to attempt cutting edge therapeutic strategies (Clemente et al., 2012).

Appropriate dietetic regimens and physical exercise may improve simple steatosis, even though this lifestyle approach is unable to recover NAFLD-associated liver damage. In fact, it is widely recognized that lifestyle modifications combined with a multi-targeted therapeutic approach against specific triggering factors could be more effective than mono-therapeutic approach in at least paediatric NAFLD (Alisi and Nobili, 2011). Unfortunately, various inadequate pharmacological therapies (e.g., insulin-sensitizers, antioxidants, and cytoprotective agents) have been developed over recent years in the attempt of modifying one or more of the major factors involved in NAFLD pathogenesis. Therefore, modifications of gut microbiota may be one of the possible objectives of an efficient multi-target therapy. This option is supported by several investigations in animal models studies suggesting that gut microbiota manipulation with probiotics reduces intestinal inflammation and improves the epithelial barrier function (Iacono et al., 2011). Furthermore, Loguercio et al. (2005) demonstrated that a chronic therapy with a probiotic (VSL#3) in patients affected by several types of chronic liver diseases, including NAFLD may reduce liver damage and improve serum levels of various biomarkers. Interestingly, a pilot study, involving 20 obese children with hypertransaminasemia and bright liver at ultrasound, found that 8 weeks of treatment with probiotic *Lactobacillus rhamnosus* strain GG reduced transaminase levels and antipeptidoglycan-polysaccharide antibodies, a surrogate test for SIBO evaluation (Vajro et al., 2011).

In conclusion, as several observations suggest a potential role of microbiota in NAFLD development, we believe that probiotics effects on gut flora associated to their excellent tolerability may represent promising therapeutic agents to revert NASH-related liver damage.
